# Happy hypoxemia, or blunted ventilation?

**DOI:** 10.1186/s12931-020-01604-9

**Published:** 2021-01-06

**Authors:** Josuel Ora, Paola Rogliani, Mario Dauri, Denis O’Donnell

**Affiliations:** 1 Division of Respiratory Medicine, University Hospital Policlinico Tor Vergata, Rome, Italy; 2 Department of Experimental Medicine, University of Rome Tor Vergata, Rome, Italy; 3 Department of Clinical Sciences and Translational Medicine, Tor Vergata University Hospital, Rome, Italy; 4 Respiratory Investigation Unit, Department of Medicine, Queen’s University, Kingston, ON Canada

**Keywords:** Dyspnea, COVID-19, Dyspnea descriptors, Respiratory drive, Hypoxemia

## Abstract

Happy hypoxemia is an unspecified definition that is used in COVID-19 patients to define hypoxemia without dyspnoea. Dyspnoea is a very complex symptom, and although hypoxemia can cause breathlessness, dyspnoea is not related to hypoxemia, but is more closely related to inspiratory drive and mechanical alterations. The lack of dyspnoea in the early stages of the disease is likely related to the absence of increased inspiratory drive due to compensatory mechanisms of hypoxemia, while in the advanced stages there is no evidence of a lack of dyspnoea in COVID-19 patients.

## Letter to the editor

We read “The pathophysiology of ‘happy’ hypoxemia in COVID-19” by Dhont et al. [1] and the related comment by Tobin et al. [2] with interest and although we agree with many concepts, there are some points that need clarification.

The authors started from the assumption that "happy hypoxemia" was clearly related to COVID-19 and then proceeded with the explanation of its mechanisms, but we think that this assumption needs stronger evidence. We too were attracted by the hypothesis that the lack of dyspnea perception was due to a neurological involvement of the nervous system in COVID-19 patients, but we could not demonstrated any relationship between lower dyspnea perception and neurological involvement of the nervous system [3]. Moreover, our data and available data on COVID-19 patients seem to confirm that dyspnea is more frequent in more severe patients, confirming a relationship between hypoxemia and dyspnea, rather than the other way around [4, 5]. Dhont et al. asserted that many COVID-19 patients have lack of dyspnea, and they quoted Tobin et al., who published a case report of 3 subjects with hypoxemia and lack of dyspnea [6], Wilkerson et al, who published another case report [7], and then two papers that only reported some anecdotal stories without any data [8, 9]. Even if there are some cases of COVID-19 patients who do not perceive dyspnea despite severe hypoxemia, as the authors affirm, this it is not exclusively seen in COVID-19, but may also occur in patients with atelectasis, intrapulmonary shunt or right-to-left intracardiac shunt, and there is no evidence that the prevalence of the lack of dyspnea perception is more frequent in COVID-19 pneumonia compared to other pneumonias, or that it is a typical feature of this condition due to the involvement of some neurological pathway [3].

Dyspnea is a complex symptom defined as “a subjective experience of breathing discomfort that consists of qualitatively distinct sensations that vary in intensity” [10]. Work/effort to breathe, difficult inspiration/air hunger and tight chest are three different well studied qualities of dyspnea and underpin distinct neurological pathways [10]. Surely, the first step for studying dyspnea is to anchor it to physiological parameters (mostly the central respiratory neural drive) or to mechanical loads such as ventilation or respiratory rate (RR) [11]. Linking dyspnea to oxygen rather than to ventilation, as in the title itself “happy hypoxemia”, can be misleading, because dyspnea is not the perception of hypoxemia, but “surprise would arise only if sensory afferents or hypoxemia elicited significant stimulation of the respiratory centers and the patient did not develop dyspnea” [6]. For example, the Wilkerons et al. patient without dyspnea had 85% of saturation, but normal RR and maybe that is the reason for the absence of dyspnea. Surprisingly, Tobin et al. do not provide data on their three patients’ respiratory parameters [6]. Although the authors are right when they say that in the Guan study the prevalence of dyspnea was 18.7% overall, they omitted that it was higher in more severe patients (37.6%) than in non-severe patients (15.1%) and also higher (53.7%) in patients admitted to the ICU [4]. Similarly, in the Huang study [5] the prevalence of dyspnea was 55% (92% in the Intensive Care Unit (ICU) patients vs 37% of non-ICU patients), and it was related to a higher RR in more severe patients.

**Fig. 1:**
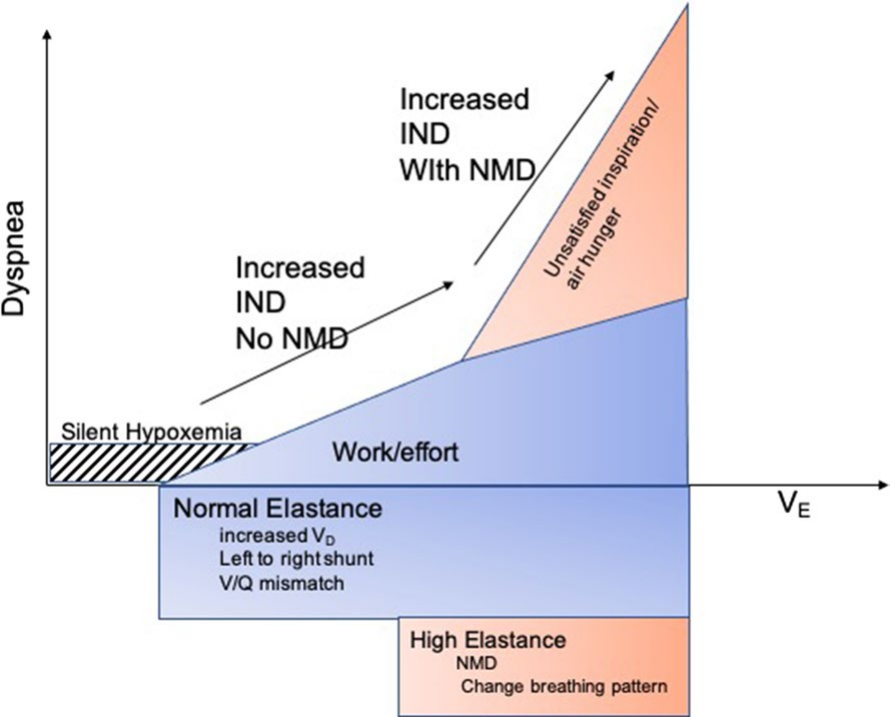
Dyspnea model in COVID-19. The increase in dyspnea correlates with the increase in ventilation. In the early stages of COVID-19 the alterations are mainly due to alterations of gas exchange and do not cause major changes in respiratory mechanics and mechanical constraints (normal elastance), with a good relationship between respiratory drive and mechanical coupling, and dyspnea will be described as increased work/effort, in the advanced stages there are alterations of respiratory mechanics with mechanical constraint (high elastance) and neuromechanical dissociation and dyspnea will be described as air hunger or difficult inspiration. If hypoxemia is not accompanied by increased ventilation, it will not cause dyspnea and it could be defined “happy or silent hypoxia”. *NMD* neuromechanical dissociation, *V/Q* ventilation/perfusion, *IND* inspiratory drive

As has been widely demonstrated [1, 6, 12], hypoxemia is only a weak stimulus for respiratory drive and consequently for dyspnea if it is not accompanied by an increase in CO_2_ or a reduction in pH, therefore more than “happy hypoxemia”, it is only a physiological blunted ventilation to the hypoxic stimulus. On the other hand, if an increased respiratory drive due to hypoxemic stimulus without any dyspnea perception had been demonstrated, then the mechanisms involved could be caused by the damage of afferent fibers due the cytokine storm syndrome of SARS-CoV2, similar to the mechanisms of ageusia and anosmia, as hypothesized by other authors [13]. Figure 1 summarizes a possible pattern of dyspnea in COVID-19 patients.

It is easier to say what we do not know about COVID-19 and dyspnea, instead of what we know. We do not know if the lack of dyspnea is a typical feature of COVID-19 or if it is similar to other pathologies, we do not know its prevalence nor its incidence, we do not have physiology studies to describe the relationship among dyspnea and respiratory drive, we do not know the quality of dyspnea described by patients and only further research can answer these questions.

## Data Availability

Not applicable.

## Abbreviations

RR: Respiratory rate
ICU: Intensive care unit
